# The Electric Lamp

**Published:** 1885-03

**Authors:** W. H. Trueman


### THE ELECTRIC LAMP IN THE MOUTH.

BY W. H. TRUEMAN, D. D. S.

I have been using a small electric lamp in the mouth a little over a month, the mounting of which is home-made. A wooden case encloses the bulb, leaving a space of one-eighth of an inch between the wood and the glass, which is packed with asbestos ; a hole three-eighths of an inch in diameter is made in the case directly opposite the carbon loop, through which the light passes. The non-conducting case was suggested by the heat developed when the lamp was in operation. It not only protects the lamp from accident, but also preserves the patient from injury should it come in contact with sensitive tissues, or accidentally break while in the mouth. Is there not some risk in using those now on the market which are either not protected at all or only imperfectly so ?

The case of mine becomes uncomfortably heated if kept in continuous use more than a few minutes at a time, while it soon produces a dryness of the mouth that is decidedly unpleasant.

Now, as to its practical usefulness. It is very convenient to illuminate the back part of the mouth on dull days, or towards evening, and a help to some extent in making examinations. It shows some defects between teeth that are otherwise difficult to see. Light can be readily thrown into some cavities which would be difficult to illuminate by any other means, and in various ways it is a handy instrument to have around.

It attracts a great deal of attention. Patients think it a sure sign that the doctor keeps "well up to the times," and is ever ready to utilize all the improvements science provides, which he thinks will add to their comfort or make his services more profitable, "regardless of expense." They talk about it; that makes it an advertisement not to be despised, and one that infringes no rule of professional ethics.

It promises to be useful in diagnosing the condition of pulps in doubtful cases, and also in detecting decay on approximal surfaces. In its incipient stages I find its assistance in these cases very slight.

Held behind the gums, especially of the anterior portion of the lower jaw, it produces a beautiful and unique effect. The tissues seem quite translucent, and aided by a little imagination, we seem to see the roots of the teeth down to their very apex. Some say that they can see perfectly the condition of the pulp, whether it is congested, dead, or dying; if the pulp canal is filled, they can see how far the filling extends, and are able to judge whether it has been perfectly or imperfectly done. This I cannot do. The tissues *seem* translucent, but they are so illuminated, or so affect the light, that it seems able to shine around obstructions. I have examined teeth before applying arsenic, after the arsenic has been in position some time, and after it has been removed and the pulp found to be dead, and have seen no difference whatever, through the gum, between the tooth in fault and adjoining teeth known to be in a healthy and normal condition. After the pulp canal has been filled, there is usually a little shading extending towards the apex of the root, but this does not extend one-third as far as the root is known to be solidly filled. Even roots with a gold pivot extending nearly to the apex do not show its presence more than perhaps one-third of its length. In some instances the opacity of the pulp canal is seen more plainly than in others, but the fact that in a notable number of cases its condition cannot be detected at all, lessens the value of the electric lamp as an aid to diagnosis. In cases where the condition of the pulp has been in doubt, it has so far proved useless, and these are the cases where aid is most needed.

In detecting approximal decay its indications are uncertain. I have found decay where the light has shown no evidence of it, and have detected it by the light where other means have failed. It *does* show the presence of tartar under the margins of the gum, unmistakably.

I would suggest, in conclusion, that all lamps for dental use should be provided with a switch in the handle, so that the lamp may be lighted only when actually needed, to prevent overheating. A convenient arrangement should also be provided for regulating the current, preferably in the handle. Regulating the intensity of the light is an important matter; many points are plainly seen when the light is rather dim that cannot be seen at all in an intense light; and many points are brought out by varying the intensity of the light during the examination.

A few inches of German silver wire, say fifteen inches of No. 28, so arranged that all or a small part may be included in the circuit, will, by its resistance, give all the latitude needed with the battery usually used for the electric mallet.

